# Study of user experience of an objective test (QbTest) to aid ADHD assessment and medication management: a multi-methods approach

**DOI:** 10.1186/s12888-017-1222-5

**Published:** 2017-02-10

**Authors:** Charlotte L. Hall, Althea Z. Valentine, Gemma M. Walker, Harriet M. Ball, Heather Cogger, David Daley, Madeleine J. Groom, Kapil Sayal, Chris Hollis

**Affiliations:** 1 Division of Psychiatry and Applied Psychology, CLAHRC-EM, Institute of Mental Health, University of Nottingham Innovation Park, Triumph Road, Nottingham, NG7 2TU UK; 2 Division of Psychiatry and Applied Psychology, University of Nottingham & Centre for ADHD and Neurodevelopmental Disorders across the Lifespan (CANDAL), Institute of Mental Health, University of Nottingham Innovation Park, Triumph Road, Nottingham, NG7 2TU UK; 3 Centre for ADHD and Neuro-developmental Disorders across the Lifespan (CANDAL), & MindTech, Institute of Mental Health, Division of Psychiatry and Applied Psychology, University of Nottingham Innovation Park, Triumph Road, Nottingham, NG7 2TU UK

**Keywords:** Attention Deficit Hyperactivity Disorder (ADHD), QbTest Activity, Objective Measures, Qualitative, Acceptability

## Abstract

**Background:**

The diagnosis and monitoring of Attention deficit hyperactivity disorder (ADHD) typically relies on subjective reports and observations. Objective continuous performance tests (CPTs) have been incorporated into some services to support clinical decision making. However, the feasibility and acceptability of adding such a test into routine practice is unknown. The study aimed to investigate the feasibility and acceptability of adding an objective computerised test to the routine assessment and monitoring of attention deficit hyperactivity disorder (ADHD).

**Methods:**

Semi-structured interviews were conducted with clinicians (*n* = 10) and families (parents/young people, *n* = 20) who participated in a randomised controlled trial. Additionally, the same clinicians (*n* = 10) and families (*n* = 76) completed a survey assessing their experience of the QbTest. The study took place in child and adolescent mental health and community paediatric clinics across the UK. Interview transcripts were thematically analysed.

**Results:**

Interviewed clinicians and families valued the QbTest for providing an objective, valid assessment of symptoms. The QbTest was noted to facilitate communication between clinicians, families and schools. However, whereas clinicians were more unanimous on the usefulness of the QbTest, survey findings showed that, although the majority of families found the test useful, less than half felt the QbTest helped them understand the clinician’s decision making around diagnosis and medication. The QbTest was seen as a potentially valuable tool to use early in the assessment process to streamline the care pathway. Although clinicians were conscious of the additional costs, these could be offset by reductions in time to diagnosis and the delivery of the test by a Healthcare Assistant.

**Conclusions:**

The findings indicate the QbTest is an acceptable and feasible tool to implement in routine clinical settings. Clinicians should be mindful to discuss the QbTest results with families to enable their understanding and engagement with the process. Further findings from definitive trials are required to understand the cost/benefit; however, the findings from this study support the feasibility and acceptability of integrating QbTest in the ADHD care pathway.

**Trial registration:**

The findings form the implementation component of the Assessing QbTest Utility in ADHD (AQUA) Trial which is registered with the ISRCTN registry (ISRCTN11727351, retrospectively registered 04 July 2016) and clinicaltrials.gov (NCT02209116, registered 04 August 2014).

## Background

Attention deficit hyperactivity disorder (ADHD) affects approximately 4–8% of school-aged children [1] and is characterised by symptoms of inattention, impulsivity and hyperactivity that is inappropriate for the person’s age. The assessment of ADHD typically relies on the clinician’s judgment which is supplemented by the integration of parent, teacher and, where age-appropriate, young person reports. These reports can be contradictory, incomplete and are often not returned in a timely manner, leading to delays in diagnostic decision making [2, 3]. The assessment of ADHD is further complicated by a high co-occurrence with other disorders, making a differential diagnosis complicated and often resulting in multiple clinic visits to obtain an accurate diagnosis [3] and substantial cost to the health services [4].

One approach to improving assessment in ADHD is to supplement clinical judgement with computerised tests completed by the child as these provide a more objective measure of attention, impulsivity and activity [2, 5, 6]. Although CPTs should not be used as a stand-alone tool for assessment, they can be integrated with other sources of information, such as parent/teacher reports and clinical opinion to reach a diagnostic decision. One CPT, ‘QbTest’ (QbTech Ltd, www.qbtech.com) is a commercially available measure of ADHD symptoms approved by the FDA (Ref: K133382) that combines a cognitive test (continuous performance test; CPT) designed to measure attention and impulse control with a high-resolution motion-tracking system to measure hyperactivity. The child/young person is seated in front of a computer and is instructed to press a hand-held responder button each time a pre-designated infrequent target stimulus appears on-screen, and to withhold the response to all other stimuli. These features of QbTest measure sustained and selective attention (target detection over 600 trials), and impulsivity (failure to withhold the response to a non-target). Simultaneously, an infra-red camera tracks the movement of a marker attached to a headband worn during the test, to measure activity. There is a substantial literature in cognitive psychology/psychiatry indicating that the CPT is sensitive to inattention and impulsivity and that the addition of the activity measure further improves sensitivity to ADHD [6]. The test provides a summary score relevant to each symptom domain (inattention, hyperactivity, impulsivity) with reference to a large age- and gender-stratified normative database (Ulberstad, unpublished). The available data on QbTest has shown good psychometric characteristics. Qbtech report data of 85% sensitivity and 92% specificity of classification [7]. Reh et al. [8] investigated the factor structure, concurrent and discriminant validity of the QbTest and found the activity measure to correlate with hyperactivity ratings provided by teachers. Additionally, research has shown that the activity measure can also identify intermediate levels of impairment in ADHD siblings [9]. Although the researchers point out the need for additional research on the validity of the test, they also found concurrent validity for the three factors (attention, impulsivity and activity) [8, 9]. QbTest has also been shown to be able to aid differentiation of ADHD from other diagnoses in children [10] and adults [11]. Research has also indicated the QbTest is a valid measure of treatment outcome [12] and effective in measuring the effects of ADHD medication [13, 14]. Audit data also suggest that QbTest may reduce the number of assessments required to reach a diagnosis [5]. However, to the best of our knowledge, there is no published research on healthcare professionals’ (HCPs) or families’ opinions on the use of objective computerised measures to aid ADHD practice.

The ‘Assessing QbTest Utility in ADHD-Trial’ (AQUA-Trial [2]) is a randomised controlled trial (RCT) which aims to determine whether adding the QbTest to standard asse ssment procedures can accelerate time to diagnosis (whilst not compromising diagnostic accuracy) when compared to assessment as usual (assessment without the QbTest report). As a secondary aim the trial also investigates whether the QbTest can improve medication titration. A further aim of the trial is to explore the attitudes of HCPs and families towards QbTest. This will elucidate potential barriers and facilitators to its use and explore stakeholders’ perspectives as to whether QbTest is considered a valuable tool that should be implemented in routine clinical settings. The protocol of the AQUA-Trial is outlined in Hall et al. [2]. The AQUA-Trial is registered with the ISRCTN registry (ISRCTN11727351) and clinicaltrials.gov (NCT02209116).

In order to assess the clinical utility of the QbTest to aid ADHD assessment and medication management in young people, we explored the perceptions of HCPs and families (parents and young people) who participated in the AQUA-Trial. There is a noticeable absence of qualitative data on the use of CPTs and other objective/computerised assessments of ADHD in the literature; however, this information is vital to inform future research trials, pathways and clinical guidelines that are designed to suit the needs of stakeholders. The aim of this study was to explore these opinions using qualitative data and a larger sample of survey data to cross-validate findings.

## Methods

### Qualitative interviews

#### Participants

Participants in the AQUA-Trial were recruited from ten sites across nine National Health Service (NHS) Trusts in the United Kingdom. Collectively, the sites combine Child and Adolescent Mental Health Service (CAMHS) and Community Paediatric services. The clinical lead for the trial at each of the ten sites was invited to interview about their experience of the QbTest and being part of the AQUA-Trial. The sites were dispersed across the north, south, east and west of England and Northern Ireland. A random selection of parents and young people who had participated in the trial was also invited to interview. All participants were approached over the phone or via email by a researcher, written consent was obtained as part of trial participation. Ethical approval was granted by NRES Committee West Midlands – Coventry and Warwick and Research and Development Department of each respective NHS Trust.

##### Healthcare professionals (HCPs)

Ten out of the 10 (100%) HCP leads (one per site) that participated in the trial agreed to be interviewed. The characteristics of the sample are displayed in Table 1. Five HCPs were already using QbTest as part of their clinical practice prior to the trial; five were introduced to QbTest as part of trial participation.

**Table 1:** Clinician characteristics (*N* = 10)

Clinician details	Response (%)
Gender (*Female*)	8/10 (80)
Profession	
Consultant Paediatrician	4/10 (40)
Consultant Psychiatrist	4/10 (40)
Nurse Specialist	2/10 (20)
Experience of ADHD	Range: 8–25 years Mean: 17 years (*SD* = 6.5)
Experience of *QbTest*	Mode: 50+ tests (70%) Range: 25 - > over 200 tests
Current methods used to diagnose ADHD:	
Developmental history	10/10 (100)
Rating scales	10/10 (100)
School observation	5/10 (50)
QbTest	5/10 (50)
Current methods to manage medication:	
Clinical opinion	10/10 (100)
Rating scales	4/10 (40)
QbTest	2/10 (20)

*Note.* Current methods to diagnose/medicate was pre-trial participation. Note. Percentage provided in parentheses

Two families per site were randomly selected for interview. Altogether, 38 families were invited to interview. Eighteen (47%) of those selected declined to participate. Refusing families were replaced with the next family until two families from each site were enrolled for interviews, totalling 20 participants (53% response rate of those invited to interview; 7.5% of the total sample [*n* = 267] recruited into the AQUA-Trial). The characteristics of the sample are displayed in Table 2. In each case, the main care-giver was the primary interviewee; however, where possible the young person was encouraged to participate in the interview with their parent. Six young people co-participated in the interview. Two families were interviewed from each site. All young people had been referred for an ADHD assessment and were in the intervention arm of the trial (received the QbTest report).

**Table 2:** Family characteristics

	Interview sample *n = 20* (%)	Survey sample *n = 76* (%)
Child gender (*Male)*	15/20 (75)	60/76 (79)
Child age	*M =* 10.7 years (*SD =* 2.9) Range = 9–18 years	*M* = 10.2 years (*SD* = 2.9) *Range* = 7–18 years
Confirmed primary diagnosis		
ADHD	11/20 (55)	35/76 (46)
Not ADHD	5/20 (25)	11/76 (14)
Unconfirmed	5/20 (25)	30/76 (39)
Co-morbidities with ADHD		
Autism Spectrum Disorder	1/20 (5)	4/76 (5)
Conduct Disorder and Oppositional Defiance Disorder	0/20 (0)	3/76 (4)
Tourette’s/Tics	1/20 (5)	1/76 (1)
Attachment Disorder	0/20 (0)	1/76 (1)
Learning Difficulties	0/20 (0)	2/76 (3)
Anxiety and Depression	0/20 (0)	1/76 (1)

*Note.* Percentage provided in parentheses

#### Procedure

Prior to the interviews commencing, all participants had read an information sheet and signed a consent form. All interviews were conducted by a trained female researcher on the trial (CLH *(PhD*), GW *(BSc*), HC (*MSc*) & HB (*BSc*)) and recorded on a Dictaphone to aid subsequent transcription. Interviews took place individually with each participant over the telephone. Participants had received some limited contact with the members of the research team as part of the trial (e.g. questionnaire completion and follow up reminders) and were informed that they were being interviewed to gain their opinion on the QbTest.

The interviews were semi-structured and guided by separate interview schedules for each participant group (HCP or families), the interview schedule was adapted for the young person. Interview schedules and survey questions were formulated prior to the main trial by the research team. The views of parents of children with ADHD, clinicians and an implementation expert was sought on draft documents prior to data collection and questions amended accordingly following their feedback. The interview schedules were amended where necessary throughout the trial to reflect informal feedback received from participants regarding their experience of the trial. By utilising a semi-structured approach, the researcher was able to ask additional questions based on the interviewees’ responses. Questions for discussion included participant’s experience of participating in the trial, their opinion on QbTest clinical utility, issues with current ADHD practice (HCPs only) and the future use of the QbTest. A series of prompts were generated for each question to aid discussion if required. Interviews ranged from approximately 20–60 min in length. Presented quotes are reflective of the views of multiple responders providing similar statements unless otherwise specified.

#### Analysis

Audio recordings were anonymised and transcribed verbatim. Transcripts were first thematically analysed by CLH (*PhD*), following the guidelines of Braun and Clarke [15] using an inductive, reflexive approach. Each coding unit was coded exclusively into just one category rather than multiple categories to create well defined coding categories [16]. To ensure validity and reliability of data interpretation, the transcripts were then second coded by another researcher (AZV, (*PhD*)). Overall themes were largely consistent between coders, where contradictory coding was apparent, the coders resolved this through discussion until consensus was reached. The coders’ epistemology was that of an essentialist/realist paradigm [15], which sought to understand the opinions of QbTest through the words of the participants, as opposed to the researchers’ co-created meaning.

### Quantitative survey

#### Procedure

All participants and HCPs in the trial were invited to participate in an online survey hosted by www.surveymonkey.com. The HCP survey questions were centred on how best to administer the QbTest, understanding the results and communicating with families. Families in the intervention arm of the trial were also invited to participate. Families in the control arm were not interviewed on their perceptions of QbTest as they did not receive the QbTest report. The parent/young person survey asked questions pertaining to the utility of the QbTest in understanding symptoms and diagnostic decisions and the experience of completing the test. Data were collected from May 2015-May 2016 and downloaded from the website at the end of the study. Descriptive statistics were used.

#### Respondents

The HCP survey was sent to each participating site and was completed by the same HCP (100% response rate) that participated in the qualitative interview (see Table 1). The survey was sent to all 132 families in the intervention arm of the trial, of which 76 (58%) responded (see Table 2 for sample characteristics). In each case, the survey was addressed to the primary caregiver and it was requested that the survey was completed by the parent and young person together (where possible). There was no difference in age (*t-*test *p* > .05) or gender (chi-square *p* > .05) of the young person between respondents in the survey and the rest of the sample. However, there was a significant difference in diagnosis (ADHD confirmed, excluded, unconfirmed; chi-square *p* < .05), with proportionately less people receiving an unconfirmed and ADHD confirmed diagnosis in the survey sample than the whole trial sample.

## Results and discussion

Three salient themes emerged from the qualitative interviews, relating to the clinical validity of the QbTest, the use of the QbTest in communication and the role of the QbTest in the ADHD care pathway. A summary of themes and associated sub-themes is provided in Table 3. The findings of the qualitative interviews were supported by the survey data which are summarised in Table 4 (HCPs’ responses) and Table 5 (families’ responses).

**Table 3:** Summary of themes

Main theme	Sub-themes
Clinical validity of the QbTest	Objectivity Understanding symptoms Further evidence
Communication and the QbTest	Communication between stakeholders Effects of medication
The QbTest in the care pathway	Time to diagnosis Barriers and facilitators (time and cost, staffing and resources, training and practice) Placement in pathway

**Table 4:** HCPs opinion on the clinical utility of the QbTest (*N* = 10)

	Strongly agree/agree (%)	Neither agree/disagree (%)	Strongly disagree/disagree (%)
Helps me understand patient symptoms	10/10 (100)	0/10 (0)	0/10 (0)
Improves patient communication	10/10 (100)	0/10 (0)	0/10 (0)
Makes it easier to explain why they do or do not have ADHD	10/10 (100)	0/10 (0)	0/10 (0)
Is not a good use of patient time	0/10 (0)	0/10 (0)	100/10 (10)
Easily incorporated into assessment clinics	10/10 (100)	0/10 (0)	0/10 (0)
Should be used routinely as part of assessment	7/10 (70)	3/10 (30)	0/10 (0)
Should be reserved for cases of diagnostic uncertainty	3/10 (30)	1/10 (10)	6/10 (60)
Best administered by HCA (or equivalent)	6/10 (60)	3/10 (30)	1/10 (10)
Best administered prior to clinicians first contact with the child	6/10 (60)	2/10 (20)	2/10 (20)
Helpful to evaluate treatment effects	10/10 (100)	0/10 (0)	0/10 (0)

*Note. HCA* = Healthcare Assistant. Percentage provided in parentheses

**Table 5:** Families’ opinion on the clinical utility of the QbTest. (*N* = reported per question)

	Strongly agree/agree (%)	Neither agree/disagree (%)	Strongly disagree/disagree (%)
Helped us to understand the symptoms (*n* = 73)	35/73 (48)	22/73 (30)	16/73 (22)
The results were difficult to understand (*n* = 72)	17/72 (24)	29/72 (40)	26/72 (36)
Fully understood the purpose of the test (*n* = 74)	56/74 (76)	12/74 (16)	6/74 (8)
Found the test stressful (*n* = 74)	17/74 (23)	24/74 (32)	33/74 (45)
The QbTest was useful (*n* = 72)	41/72 (57)	18/72 (25)	13/72 (18)
Helped to understand how the diagnosis was made (*n* = 68)	31/68 (46)	23/68 (34)	14/68 (20)
Helped us to understand the decisions on medication (*n* = 52)	20/52 (39)	24/52 (46)	8/52 (15)

*Note.* Percentage provided in parentheses

### Theme 1 – clinical validity of QbTest

#### Objectivity

HCPs and families commented on the perceived validity of the QbTest. Of particular importance to both groups was that the QbTest report provided an objective, observable measure of symptoms. Given the current reliance on subjective approaches to diagnose ADHD [2, 6] it appears that clinicians and families alike would welcome a more objective measure.

“I think to be able to see something, it’s that black and whiteness of it, to look at it and go yeah I can see that” (Parent 8).

HCPs noted that the objectivity of the QbTest report encouraged them to feel more confident in their decision making.

“I would move to the diagnosis more confidently and more quickly having evidence that something was wrong, you know objective evidence. …reduced the amount of the anxiety of uncertainty” (HCP 1).

The QbTest was also seen as a clinically valid tool to objectively assess medication effects. All clinicians reported that the QbTest was helpful to evaluate treatment effects and 39% of families surveyed felt that it helped them to understand the decisions on medication. The uncertainty reflected by families may be because not all participants commenced medication during the trial (approximately 75% not medicated) and parents responded ‘neither agree/disagree’ or ‘strongly disagree/disagree’ when in fact they had not been prescribed medication (see also Tables 4 and 5). HCPs reported that their increased confidence in their diagnosis often led to speedier initiation of medication.

“I think that I was probably quicker to agree to initiation of medication when I was able to do the QbTest, I think it probably gave parents maybe more confidence” (HCP 4).

A thought shared by families during interviews:

“It’s a big decision to allow your children to have these drugs, as it were. So, again, seeing those results made me more confident that yes the medication would help him” (Parent 17).

#### Understanding symptoms

All clinicians felt that the QbTest helped them to understand symptoms (see Table 4), which was particularly valuable in complex cases. In contrast, only approximately half of the survey families also felt it helped them understand their child symptoms better (see also Table 5). Further detailed analysis from the qualitative interviews showed that whereas some families felt the clinician had discussed the report fully with them, others did not (see Theme 2 – communication). In instances where parents felt it had helped them understand their child symptoms and behaviours, parents sometimes reported an improved parent/child relationship.

“I only see what he’s like at home so it was nice to see what he has done [in the QbTest] to help me understand it” (Parent 3).

In general, HCPs felt the QbTest was accurate. However, some noted that, at times, the test contradicted their clinical opinion. HCPs reported false negatives occurring when they felt the child was highly motivated to do well in the task (i.e., they enjoyed computer tasks or did not want a diagnosis), and false positives in the cases of children who had experienced trauma.

“A couple of cases where the Conners’ [questionnaire] and the clinical observation was ADHD, but the Qb Test wasn’t. Mainly in girls of a certain age group and I also know these girls …and they try their best and they don’t want a diagnosis” (HCP 6).

“What I would call traumatised children …they will invariably pop up on a Qb test as looking like ADHD” (HCP 3).

In line with this, some interviewed families commented that a QbTest alone was not in depth enough to provide a diagnosis, and should be used in conjunction with other measures.

“I just think there should be more, more than that to diagnose them if you know what I mean?” (Parent 1).

This supports the British Association for Psychopharmacology (BAP, [17]) guidelines that one test alone should never be used to diagnose ADHD and the FDA approval for QbTest not as a ‘stand-alone’ test; a point that was also supported by our HCPs.

“We all know that they have their limitations. They are not perfect and they never should be the only element –but they have a place” (HCP 9).

Some HCPs commented that it was important to observe the test in order to assess the validity of the results. Qbtech advocate that a trained professional is present at all times during the test and that any noteworthy behaviours or disturbances are recorded on an observation form.

“If you’re not aware of what’s actually happening at that time, then I think it might be difficult… the actual observation, what’s happening during that time, is very important” (HCP 2).

As ADHD is frequently co-morbid with other disorders, this often further complicates the clinical picture and makes interpreting reports or scales more difficult. HCPs differed as to whether they felt QbTest was useful in understanding co-morbidities or made a differential diagnosis more complex. It appeared that HCPs with greater experience of QbTest (those using QbTest prior to the AQUA-Trial) were more positive in its ability to help with co-morbidities than those with less experience.

“The reports were more complex to understand and thus required as much clinical scrutiny as without the test” (HCP 10).

In recognition of this impact of experience, Qbtech offer clinical guidance to HCPs to aid their test interpretation, particularly for complex cases. HCPs found the test useful in terms of comorbidities, primarily reporting the test aids the discrimination of Autism Spectrum Disorder (ASD), which some describe as producing in a different error pattern. In support of this, a recent study in adults demonstrated adding the QbTest to standard rating scales significantly improved the differentiation of ADHD from ASD in adults [11].

“I very often use it for children that I suspect have got ASD comorbidity. I think it’s very clear that there’s a group of children with just pure ADHD who do a Qb Test in one way, and then the group that’s got some degree of autism or autistic traits do it very differently, and I think that’s really helpful” (HCP 2).

Other differential diagnoses that HCPs reported being able to discriminate with the aid of QbTest included anxiety (typically reporting improved performance throughout the task), depression (slow processing) and learning difficulties (problems understanding the test). HCPs commented that the test was useful in differentiating ADHD subtypes. Interestingly, there was no consensus as to which symptom domain (attention, impulsivity or activity) was particularly valuable, with each being cited as important by different respondents. In line with this, a recent systematic review suggests that a test that objectively measures all three domains may be the most clinically useful [6]. However, specifically, HCPs commented on the utility of the attention measure for inattentive sub-type girls which can be hard to diagnose, supporting previous research that ADHD may be under-diagnosed in girls [18, 19], several HCPs specifically commented on the utility of this tool for measuring attention in girls.

“It was the attention aspect of it about it really helped, especially in girls…People don’t notice it because they are sitting there quietly, daydreaming” (HCP 6).

#### Further evidence

Overall respondents were supportive of the clinical validity of the test. However, in support of Hall et al. [6], some reported the need for more published data to support their clinical experience of the test.

“I think having more published research around the accuracy of Qb would be helpful” (HCP 10).

Some HCPs and families also questioned the validity of the clinical setting of the test. As such, there is need for future research to further investigate the clinical and ecological validity of the test.

“Perhaps bringing a child into a clinic, giving them a nice structured environment is not typical of what happens in school” (HCP 1).

“He behaves differently at home and school to what he would do in a clinical office sort of thing… And of course for that twenty minutes that he was seen he was on his best behaviour” (Parent 4).

### Theme 2 – communication

#### Communication between stakeholders

HCPs and families particularly valued the QbTest report to aid communication. All HCPs reported in the survey that the QbTest helped improve patient communication, however, approximately a quarter of families found the results were difficult to understand (Tables 4 and 5). HCPs valued being able to show an objective piece of evidence that they could discuss with families, particularly the comparison with the normative sample and the easy to understand graphs plotting the child’s performance across the three symptom domains of ADHD. Previous research has also shown that improved communication between HCPs and families is one of the key benefits of objective, graphical reports [2, 20].

“That [the graphs] is one of the key areas in terms of feedback … it’s quite hard to put into words actually how much of an improvement that makes, being able to visibly show something to parents. And, actually, young people” (HCP 3).

In particular, all HCPs valued the QbTest to explain why they had ruled out a diagnosis of ADHD (Table 4). HCPs reported this can be contentious, with families often refuting this diagnostic decision. HCPs felt that being able to show the families a comparison of their child’s performance to a normative sample, relatively free from interpreter bias helped families accept this decision.

“A lot of parents who previously would have probably shouted and screamed at you for not saying their child had ADHD will accept it if the computer is not showing the evidence” (HCP 2).

In general, survey results showed that families fully understood the purpose of the QbTest (Table 5) and, in the interviews, families stated HCPs explained the reports well, with parents commenting the reports were easy to understand.

“He explained it as well, he didn’t just hand it over, you know, he sort of said ‘well look, this is what this means’…so he interpreted it for us” (Parent 14).

However, in some instances families that were interviewed reported HCPs not explaining the report, or how the report was used to inform the clinical decision making, this is likely to be reflected in the survey findings that only approximately half of the families felt that the QbTest helped them to understand how the diagnosis was made (see Table 5). As a consequence of this, some families to felt that the decision had been made solely on the QbTest report, or conversely, disappointed when the HCP did not conclude a definitive diagnosis after the child had taken the test. The survey findings indicate that those who received a diagnosis of ADHD appeared more likely to view the QbTest as useful and helpful for understanding how the diagnosis was made than those who did not receive an ADHD diagnosis. This may reflect clinicians reporting that families are often disappointed or unhappy when a diagnosis of ADHD is not made. HCPs should ensure they clearly explain the findings of the QbTest report and how this informed their clinical opinion to improve families understanding of the test.

“I don’t know if she explained, it felt like the QbTest had said it so that’s what we’re going with” (Parent 8).

Parents also reported that the QbTest helped them communicate with their child, and in some cases also helped the child gain more understanding and acceptance about their own symptoms. This supports previous findings that computer-generated graphs plotting children’s symptoms can improve communication for families accessing child mental health services [21].

“He was quite happy that he scored so highly on it! In the sense of ‘see mum, it’s not my fault’ …there’s a reason to this, it’s not just me being immature, pain in the backside, whatever” (Parent 17).

During the interviews parents and HCPs commented that the reports can also improve communication with schools. Particularly in cases where there was a parent/school conflict as to whether the child had a diagnosis of ADHD.

“I’ve got something else to back me up with in what I was saying … because I can obviously say its hyperactivity…but that test also its written proof, not just my word” (Parent 11).

“And to discuss it with teachers who don’t see a problem, when you’ve given them a really detailed report showing exactly why there is a problem” (HCP 5).

#### Communicating medication effect

HCPs reported the QbTest was useful for helping communicate the effect of medication to families, something that families can find difficult to judge. In support of this, families participating in the qualitative interviews commented that the QbTest helped them understand the effect the medication was having, however, Table 5 shows that some survey respondents were less certain. This may be partly explained by not all respondents having experienced their child perform a QbTest on medication. Previous research has identified that young people taking ADHD medication would value a graphical representation showing the effect of medication [20].

“Not everything you can see, if you do QbTests you can actually see what they’re doing and then it’s all printed out on paper and you can tell whether they’re getting better” (Parent 5).

HCPs also noted that families can often have an unrealistic expectation of the effect of medication and the QbTest report can be particularly useful for demonstrating the ADHD behaviours are improving, and any ‘undesirable’ behaviour may actually be typical childhood behaviour, or require a different intervention strategy (such as parenting programmes).

“The medication’s working well, it’s really helpful to have a bit more objective to say ‘look, actually this isn’t necessarily to do with ADHD and it could be to do with something else” (HCP 8).

In addition, HCPs commented that the report can be used to resolve conflict and debate within families or with schools as to the benefit or effect of medication.

“I've used it also in medication titration to evidence where there is a clear difference on and off treatment with acrimonious parents who can’t agree” (HCP 4).

As a result of being able to directly observe the effect of medication, HCPs reported greater support from parents on initiating and continuing medication, and greater adherence to medication. Non-adherence to ADHD medication is frequently reported [22–24] and may explain suboptimal outcomes for young people with ADHD [23, 25].

“They can see there is a difficulty there and that the medication can improve that, I think it does really improve adherence and understanding of what the difficulties are for the kids more than anybody else” (HCP 1).

### Theme 3 –QbTest in the care pathway

A pivotal theme that emerged from the data was the potential role of the QbTest to streamline the care pathway. Data from the AQUA-Trial showed that many referrals are not diagnosed within six months of their first appointment. Delays in receiving ADHD diagnosis have been previously reported [21] and may result in increased health service and government expenditure as a result of time off work and school to attend additional clinic appointments. HCPs felt that QbTest could help reduce delays in diagnosis and treatment onset, a finding supported by a recent audit study [5]. Here, we present key factors that should be considered in creating adding QbTest to the ADHD care pathway.

“You can see that some people are much more confident with early diagnosis, use different preparations, different titrations… and I think that one thing would be just insist that it [the care pathway] incorporates something like a Qb, we’ve got to have a lot more robust pathway and that wouldn’t be a bad thing!” (HCP 4).

#### Time to diagnosis

Given pressures from service funders, commissioners and providers, it is not surprising that HCPs were overwhelmingly supportive of a quicker time to diagnosis with fewer appointments. However, families were more divided. Many quotes from families interviewed discussed the need for a quick decision in order to facilitate early treatment initiation, particularly for children who were struggling in education. Families also commented that going through the diagnostic procedure can be emotionally overwhelming for parents and children alike and there was no gain in prolonging this.

“Because time’s precious in the education, you know in your child’s education, well kids like this are missing out all of the time” (Parent 19).

However, where many parents wanted to see an ‘efficient’ service, a minority felt concerned that ‘labelling’ a child should not be done quickly and the HCP should spend time understanding the child over multiple appointments.

“I just wished it were more like I say I was in and out, just wished it were more appointments and a bit more time” (Parent 1).

In line with this, interviews revealed families were divided as to whether they wanted multiple short appointments or longer appointments with their HCP. Some felt the QbTest was so brief it was best to be combined with a clinician appointment to reduce visits, whereas others felt a separate appointment allowed time to reflect on the results.

“No I think the separate appointment…having time to process it and discuss it with him so he had time to understand what it was he was going to do was good” (Parent 8).

#### Barriers and facilitators

*Time and Cost:* One of the most frequently occurring topics involved the time and cost balance of the QbTest appointment. Families felt that the test was quick and as a result felt it was time well spent. Survey data also support that the majority of respondents felt the test was useful (Table 5).

“No, I think it was a good use of time… And it didn’t take long, we were in and out within half an hour” (Parent 8).

HCPs commented that although the QbTest subsumes its own time, given the objectivity of the measure it can reduce the duration of the overall decision making process, resulting in a quicker time to diagnosis.

“It has been helpful for me to shorten the diagnosis process, although it adds one appointment, but…it shortens the doubts…the being unsure” (HCP 9).

Furthermore, HCPs reported that families were happy to complete the test, which added to the value of conducting it. However, some HCPs commented that although they felt the QbTest was worth the cost, they were unsure that the health service would fund it.

“I’d like to advocate for that [QbTest as routine practice]. I’m not convinced the Trust would pay for it but I would like it” (HCP 10).

Many felt that a commissioned service would be beneficial to helping use QbTest, and that commissioners would want to see evidence of a reduction in costs in order to commission it.

“I think if commissioned would be ideal, because there’s always the cost…and I think having it in NICE [National Institute for Health and Care Excellence], having it, approved in that way because CCGs are very pedantic about what they will and will not fund” (HCP 1).

Interestingly, a recent audit has shown that the addition of the QbTest to standard practice actually reduces the cost of ADHD assessment by 20% [5]. Specifically, some HCPs felt time/cost savings could be made by replacing the lengthy and difficult to access school observations with the QbTest.

“What we did was because of QbTest results, I then stopped the school observations, so then we could confirm the diagnosis and go ahead with the medication” (HCP 2).

*Staffing and resources:* One factor that influences the time and cost is who administers the task. The majority of HCPs felt the QbTest was best administered by a trained healthcare assistant (HA) or equivalent, and then interpreted by the clinician (see Table 4).

“Healthcare assistants are much more cost effective compared to [clinicians] in delivering this test” (HCP 10).

Some clinics had a dedicated room for QbTest, those that did not advocated that a QbTest room where the equipment could be left set-up would reduce set-up time and facilitate the routine use of the test.

“So that was really helpful was a nice room, we had it all set up so it didn’t mean that we had to take it from one place to, we go in, we do the Test, it means that we are not wasting our time by setting it up and it all worked really well” (HCP 6).

Some clinics cited difficulties with accessing internet connections to upload and download the results to the QbTest website; others mentioned the difficulties in lack of access to a printer to print off the reports to discuss with the families.

“There was a lot of IT [Information Technology] governance issues to get it set up” (HCP 4).

“There are logistical problems that emerge around the fact that we haven’t always got a printer to print out the results and having it on paper is extremely helpful” (HCP 10).

*Training and practice:* HCPs reported training and multiple use of the QbTest as key facilitators to the use of the test. Qbtech provides training to all clinicians upon installation of the QbTest, which includes discussions, demonstrations and practice tests. Qbtech clinical advisors are available to offer additional test interpretation support when required. The help and support from their team received much praise.

“I think the training was really good …also what was really helpful was being able to ring up when I had test results that I wasn’t sure about … I think I would have struggled to use it without that support” (HCP 8).

#### Placement in pathway

The majority of HCPs and families interviewed felt that QbTest was best conducted prior to an initial appointment with the clinician (see also Table 4 for HCP survey response). HCPs felt that in an ideal scenario QbTest would form part of the work-up before they saw the child, to enable them to have a better understanding of the child’s symptoms at the first appointment and facilitate quicker decision making.

“I would then also even put a QbTest in as a precursor to the initial consultation so that at the time you see the child, they’ve had all the relevant questionnaires completed from home and school and a QbTest and you could probably make a diagnosis on the first appointment” (HCP 4).

Likewise, families were also supportive of this, with one suggesting it would be best placed in GP surgeries as part of an initial screen before referral to specialist services.

“You could have it available at the doctors, so you know if you’re going to your GP…to say you know we’re not sure what’s going on here…then it makes something available there” (Parent 16).

This idea was supported by HCPs who felt it would enable children to have access to the most appropriate service in a timelier manner, avoiding the lengthy and unnecessary delays often seen in child mental health settings.

“Actually having a more streamline service …rather than someone sit on a waiting list for kind of eighteen months, they finally come into you and you go ‘well this isn’t ADHD’ and then they go off to some other list!” (HCP 3).

However, HCPs were divided as to whether the test should be used on all cases as part of diagnostic assessment (see also Table 4). In an ideal world, HCPs felt using QbTest on all cases would be beneficial, due to its objectivity, comparison to a normed sample and ability to increase diagnostic confidence. However, within real-world constraints, many felt that in straight-forward cases the QbTest would not be necessary.

“Well, I would say it was financial constraints but certainly time constraints and, do we actually need to be spending that sort of money and that time or that person delivering the tests, etcetera and reviewing the results when it’s straightforward enough?” (HCP 5).

As discussed previously, there was unanimous agreement from HCPs that QbTest was particularly useful in complex and co-morbid cases, which was also cited as a pivotal reason for delay in ADHD diagnosis. This suggests QbTest could play a key role in facilitating diagnostic decisions in complex cases. HCPs also valued the test for providing another measure of medication effectiveness. HCPs particularly reported the role of the test to aid titration, often reporting that QbTest results led to speedier titration and also stopping at a lower dose.

“But we saw the results of only being on the lowest dose of a medication was …he’s brought his inattention to zero…. So that actually said, ‘OK, that’s enough. I’m not going to change it” (HCP 5).

Dissatisfaction with delays in medication initiation and lengthy titrations has been previously reported by HCPs and families [3, 20], as such, QbTest may prove a useful tool to facilitate prescribing medication in this process. Although the utility of the QbTest to monitor medication effects was unanimous in the survey, some interview respondents felt that in simple cases, QbTests on medication was not necessary and should be reserved only for more complex cases or parental/school queries about the effects of medication. This was partially driven by concerns on cost and time.

“[The effects of], medication is so clear to most people that it [QbTest] is only needed in those [cases] where you're not sure or whether there’s lots of complexities about the co-morbidity or the opinion” (HCP 4).

Interview data showed that families felt that having a QbTest on medication was beneficial to assess the effect of medication. In relation to this, some families also commented that they felt abandoned by the service post-diagnosis. Those that received medication reported they should have been more closely monitored, and those that were not medicated were unclear of what options were available to them.

“Like I just feel like maybe my child by the doctors and stuff has been let down a bit by not being seen and just like he said he should have been seen really after the medication and he hasn’t” (Parent 3).

Previous research has also demonstrated that families report dissatisfaction with medication monitoring [3]. The QbTest may provide a key role in providing a more robust monitoring system that encourages parents to feel more supported. However, as previously discussed it is important for HCPs to fully discuss the report to ensure that families understand diagnosis and medication decisions.

### General discussion

By gathering the perspectives of HCPs and families, both through survey data and qualitative interviews, we have obtained a unique insight into the feasibility and acceptability of incorporating the QbTest as part of practice in routine health service settings in the UK. Overall, clinicians were very positive about the QbTest in both the survey and interviews. However, although the responses from interviewed families typically were in agreement with clinicians, there was more heterogeneity found in the larger sample of families who responded to the survey.

Our findings identify a number of advantages in using QbTest, including the integration of an objective measure into an area which currently relies on clinician judgment for diagnosis, an increase in clinician confidence in decision making, perceived speedier diagnostic decisions and medication initiation, improved understanding of symptoms for both families and HCPs and improved communication for multiple stakeholders. However, survey results showed some families did not find the test useful in understanding symptoms. Combined with some families reported concerns on how the test was used to inform the clinician’s diagnostic decision, it is clear that HCPs need to ensure they fully explain the use of the test to the families to improve understanding. Respondents were positive about the use of the QbTest in the ADHD care pathway. However, these benefits need to be offset by the costs of the test. HCPs voiced concern that service commissioners would be unwilling to fund a test that in itself does have an associated cost. Interestingly, this unwillingness would be unlikely in areas of physical healthcare. To balance this, HCPs cited some ways to reduce costs including replacing lengthy school observations with QbTest, using QbTest on selected, complex cases and having the test conducted by a HA. A potential care pathway emerged from the findings, in which QbTest was conducted by a HA prior to first contact with the clinician, allowing the clinician to make speedier informed decisions and potentially reducing the time to diagnosis. Recent audit data [5] demonstrated a 20% cost reduction in diagnostic assessments for ADHD after a Trust had implemented QbTest. Clearly, further research is needed and the results from the AQUA-Trial [2] will provide further evidence on the cost/benefit on the test.

Although families were supportive of the use of QbTest to aid medication management there was greater divide amongst HCPs as to its cost-effectiveness. Further research should investigate the role of the QbTest in monitoring. Additionally, future research could investigate whether conducting the test at home or in school may prove the most economical and ecologically valid setting.

This is the first research that evaluates the views and perceptions of HCPs and service-users on the feasibility and acceptability of adding a computerised objective measure to aid ADHD practice in routine health service settings. The findings could be used to support the integration of other similar technological devices to aid practice across other conditions. Our research is strengthened by the inclusion of families and HCPs and the multi-site nature of the research, which was conducted in 9 NHS Trusts (10 sites) covering CAMHS and Community Paediatric sites across the United Kingdom. Additionally, our research is strengthened by our sample characteristics including HCPs from different professional backgrounds, with different lengths of experience of QbTest (50% new to QbTest, 50% experienced prior to the trial) and families that had received a confirmed ADHD diagnosis and no diagnosis. However, it may be that HCPs or families that were particularly motivated or interested in objective measures participated in the study. Furthermore, only approximately half the families invited to participate responded to the interview/survey, it is possible that families who viewed the QbTest positively were more likely to respond than those who did not. This potential response bias should be considered when assessing the results. Few young people participated in the interviews; as such the results are more reflective of parent beliefs than young people. Additionally, our sample size contained proportionately less people with a confirmed ADHD diagnosis or unconfirmed diagnosis than the whole AQUA-Trial sample. Although we would consider it likely that those with a confirmed diagnosis may view QbTest more favourably, equally those with an unconfirmed diagnosis may have more negative views of the test. Furthermore, the perceived benefits of QbTest need to be supported by the results of a more definitive randomised controlled trial investigating time/cost savings.

## Conclusions

The findings provide a valuable first insight into how clinical staff and families view the addition of the QbTest to aid the assessment and monitoring of ADHD. In doing so, we specifically highlight the potential use of the QbTest to streamline the care pathway and potentially facilitate speedier, more accurate diagnosis and earlier treatment initiation. The QbTest plays a key role in facilitating communication between families, professionals and schools and may result in increased service satisfaction. A number of clinical observations require further exploration, such as the role of QbTest in differentiating co-morbidities and medication adherence. We advocate the need for definitive trial findings to support these results and conclude that the findings from this study strongly support the integration of QbTest in the ADHD care pathway.

## Abbreviations

ADHD: Attention Deficit Hyperactivity Disorder
AQUA Trial: Assessing QbTest Utility in ADHD Trial
ASD: Autism Spectrum Disorder
BAP: British Association for Psychopharmacology
CAMHS: Child and Adolescent Mental Health Service
CCGs: Clinical Commissioning Groups
CPTs: Continuous Performance Tests
FDA: U.S. Food and Drug Administration
HA: Healthcare Assistant
HCPs: Healthcare Professionals
IT: Information Technology
NHS: National Health Service
NICE: National Institute for Health and Care Excellence
NRES: National Research Ethics Service
